# Medical Gymnastics for the Phthisical

**Published:** 1881-05

**Authors:** 


Medical Gymnastics for the Phthisical.—We are glad to find it briefly stated in an exchange, that Dr. Drachman, of Copenhagen, has given his experience in the use of gymnastics in developing the chest capacity, and consequently the lungs, in phthisical subjects and those predisposed hereditarily, and otherwise, to the great destroyer of the white races of men. We have for many years been able by properly arranged gymnastic manœuvers in young persons predisposed to consumption, to correct or change abdominal into thoracic breathing, and consequently to expose a larger portion of lung surface to the process of oxygenization and decarbonization of the blood. The means or appliances for this purpose need be only of the simplest character, and so easy of construction as to be within the reach of the most moderate circumstances. Thus, a round piece of wood two feet in length and two and a half inches in diameter, suspended by a strong cord in the middle to the ceiling of the bedroom or other convenient apartment, and another of similar shape and size attached in like manner by a cord or strong rope, five or six feet long, to the wall or a post, four or five feet from the floor, are the only appliances or apparatus necessary to expand the chest and change abdominal to thoracic respiration. The method of using them is exceedingly simple and at the same time philosophical in action.

On arising from bed, and if convenient during the day, the bar suspended from the ceiling should be seized with both hands, and the body drawn up until the chin is on a plain with the bar, and the movement repeated once or twice at first. The hands should then be reversed on the bar, and with their palmer surfaces looking backward, an effort should be made to suspend the body until the occiput is on a horizontal plain with the support or bar. These movements should be repeated but a few times at first, and increased as the muscles of the arms and chest become more and more accustomed to the strain upon them. The bar should always be suspended within easy reach of the person or persons for whose use it is intended. The one attached to the wall is for exercising the muscles on the front and rear of the chest, by seizing it with both hands and swinging the body against it, both anteriorally and posteriorally, several times morning and night. These methods will be found to expand the chest and develop the muscles attached to it, including the intercostals and the diaphragm, even. As nature does not willingly tolerate a vacuum, and the methods just described, when properly made, tend directly to expand the chest from base to apex, it will be readily comprehended that the practice of them must necessarily cause the air to enter every pervious portion of the lungs, and by thus enlarging the capacity of the air cells, increase the surface for the physiological action of the atmospheric air on the mucous membrane of the lungs.

Any other method, such as seizing the top of a door or window, within easy reach, and swinging the body therefrom, may be resorted to for stretching the muscles and expanding the chest, etc. But these will readily suggest themselves to the medical adviser in cases where the contrivances already named cannot be easily obtained. This physical treatment may be also advantageously applied as an adjunct to other means in cases of consumption, after the suppurative stage has developed, and even after cavity has been formed. Such mechanical means, with climate, proper regimen and appropriate medication, render tuberculosis a curable disease.

We learn that Prof. J. W. Holland, M. D., has succeeded the lamented Professor Cowling in the editorship of the *Louisville Medical News*. The mantle has fallen upon worthy shoulders, and we heartily welcome brother Holland into the editorial ranks, and wish for him and the *News* a long and prosperous career.

